# Management of recurrent end-stage achalasia with robotic-assisted esophagectomy: a case report and literature review

**DOI:** 10.1097/MS9.0000000000002640

**Published:** 2024-10-11

**Authors:** Faizan Khalid, Sana W. Augustine, Satvir Singh, Rohab Sohail, Tooba Hashmi, Mahboob Younus Shaik, Ummul Asfeen, Tamer Zahdeh, Aadil Mahmood Khan, Mansi Singh

**Affiliations:** a King Edward Medical University, Lahore, Pakistan; b Liaquat University of Medical and Health Sciences, Jamshoro. Sindh, Pakistan; c Good Samaritan Hospital-Bakersfield, Bakersfield, California, USA; d Quaid-e-Azam Medical College, Bahawalpur, Pakistan; e Dow Medical College, Karachi, Pakistan; f Deccan College of Medical Sciences, Hyderabad, India; g Internal Medicine, New York Medical College, Saint Michael’s Medical Center, Newark, New Jersey, USA; h Internal Medicine, Hadassah Medical Center, Jerusalem, Israel; i Department of Trauma Surgery, OSF Saint Francis Medical Center, Peoria, Illinois, USA; j Bogomolets National Medical University, Kyiv, Ukraine

**Keywords:** atrial fibrillation, fundoplications, laparoscopic Heller myotomy, per-oral endoscopic myotomy, pleural effusion

## Abstract

**Introduction and Importance:**

Achalasia, an uncommon esophageal motility disorder, presents therapeutic challenges, especially in refractory cases with a history of multiple surgeries. Here, we present a complex case illustrating the dilemmas and multidisciplinary approach required in managing such patients. This case underscores the relevance of newer techniques like robotic-assisted esophagectomy in refractory achalasia management.

**Case Presentation:**

A 53-year-old male with recurrent achalasia endured persistent dysphagia, reflux, and esophageal spasms despite undergoing Heller myotomies, fundoplications, and hiatal hernia repairs. Imaging revealed severe esophageal dilation and anatomical alterations post-surgeries. Opting for a robotic-assisted thoracoabdominal esophagectomy due to relentless symptoms, the patient faced technical hurdles due to adhesions and a dilated esophagus. Post-surgery, complications like thoracic duct injury, milky pleural effusion, atrial fibrillation, and limb ischemia arose, necessitating multidisciplinary intervention.

**Clinical discussion:**

Managing refractory achalasia poses significant challenges, particularly in extensively operated patients. Despite aggressive surgeries, debilitating symptoms persisted, emphasizing the need for a multidisciplinary approach. Complications like thoracic duct injury and atrial fibrillation further complicate management, highlighting the intricacies of such cases. Careful consideration of surgical options and the potential of newer techniques like POEM are crucial in navigating such complexities.

**Conclusion:**

Managing refractory achalasia, especially in patients with extensive surgical histories, requires a multidisciplinary approach and careful consideration of treatment options. This case underscores the evolving landscape of achalasia management and emphasizes the potential benefits of newer techniques like POEM in select cases.

## Introduction

Highlights

- Achalasia, a rare esophageal motility disorder, poses challenges in management.
- Laparoscopic Heller myotomy with Dor fundoplication is a widely accepted surgical approach for achalasia.
- This case report presents a successful management of achalasia using laparoscopic Heller myotomy with Dor fundoplication.
- The case is accompanied by a postoperative leak, highlighting a rare complication and its management strategies.
- A comprehensive literature review supplements the case report, providing insights into current trends and outcomes in the management of achalasia.

Achalasia is an uncommon disorder with an incidence of 1.6 cases per 100 000 people^1^. It is caused by the selective loss of esophageal inhibitory neuronal function in the lower esophageal sphincter, leading to LES relaxation^2^. As a result, there is a reduction in the peristaltic power of the esophagus, leading to a functional obstruction of the GEJ^3^. The lower esophageal sphincter is hypertensive in less than 50% of patients. Laparoscopic Heller myotomy with Dor fundoplication is the standard surgical treatment for esophageal achalasia^4^. Although per-oral endoscopic myotomy (POEM) is becoming the mainstay of treatment for achalasia after gastric surgery, laparoscopic Heller myotomy with Dor fundoplication is also an effective strategy. POEM, a ‘state-of-the-art’ procedure for minimally invasive surgery, holds great promise for the future management of achalasia^5^. However, there are few reports on the use of this method after gastric surgery. Radical treatment for achalasia is currently unavailable, and most palliative procedures are designed to improve the passage of food through the gastroesophageal junction and thereby alleviate symptoms. Drug therapy is of limited and transient effectiveness. Pneumatic dilation (PD) is considered superior to endoscopic botulinum toxin injection (EBTI)^6^. Definitive conclusions regarding the benefits and risks of currently available treatments for achalasia must await the accumulation of evidence from well-designed clinical trials. We report a case of a 53-year-old man who underwent a series of laparoscopic Heller myotomy with Dor fundoplication for achalasia that remains refractory to all types of treatment. Subsequently, the decision was made to carry out a robotic-assisted thoracoabdominal esophagectomy with gastric pull-through and jejunostomy tube placement. The postoperative course was complicated with a Clavien–Dindo classification of grade IIIb. This included thoracic duct injury, milky pleural effusion, atrial fibrillation, and limb ischemia. This case report has been reported in line with the Surgical CAse REport (SCARE) guidelines^7^.

## Case presentation

We present the case of a 53-year-old Asian male who initially sought medical attention due to a constellation of distressing symptoms. The patient reported experiencing daily symptoms, including dysphagia with a sensation of food becoming stuck, choking episodes, frequent reflux, regurgitation, esophageal spasms, chronic nausea, and occasional white vomiting upon waking up. As a consequence of his ongoing symptoms and difficulty swallowing, the patient’s dietary intake was severely limited. He could only consume one meal daily, primarily soft foods. This dietary restriction significantly impacted his ability to maintain his weight, potentially further compromising his health.

The patient’s medical history was significant for end-stage refractory achalasia. To address his refractory achalasia, the patient underwent a series of surgical procedures over the years in an effort to alleviate his symptoms and improve his quality of life. These procedures included a robotic Heller myotomy, Toupet fundoplication, primary repair of hiatal hernia, redo robotic Heller myotomy with distal extension, extensive lysis of adhesions (LOA), revision Toupet fundoplication, left upper quadrant gastropexy, and a redo para esophageal hernia repair. In addition to his medical history, the patient had a history of heavy smoking, initially starting at half a pack a day at the age of 22. However, he had managed to reduce his smoking habits to a few cigarettes a day. Additionally, he reported using marijuana and occasionally consuming beer.

Despite this extensive surgical history aimed at managing his refractory achalasia, the patient’s symptoms persisted and showed a poor response to nifedipine treatment. This presented a significant challenge in achieving effective symptom relief and improving the patient’s overall well-being.

His physical examination and vital signs, however, were within normal limits. The patient completed a nuclear stress test and pulmonary function tests (PFTs) a few months prior to his admission. His nuclear stress test was unremarkable, with a left ventricular ejection fraction of 62%. His PFTs were notable for a forced expiratory volume in 1 second (FEV1) of 73% and a predicted diffusing capacity of the lungs for carbon monoxide (DLCO) of 48%. Afterward, he underwent imaging tests to assess the severity of his achalasia. Computed tomography (CT) of the chest with contrast showed stable findings of severe dilation of the esophagus with mild circumferential wall thickening of the distal esophagus and high-density material adjacent to the gastroesophageal junction reflecting postsurgical changes. Additionally, it showed layering intraluminal debris and contrast within the mid to distal esophagus.

**Figure 1:**
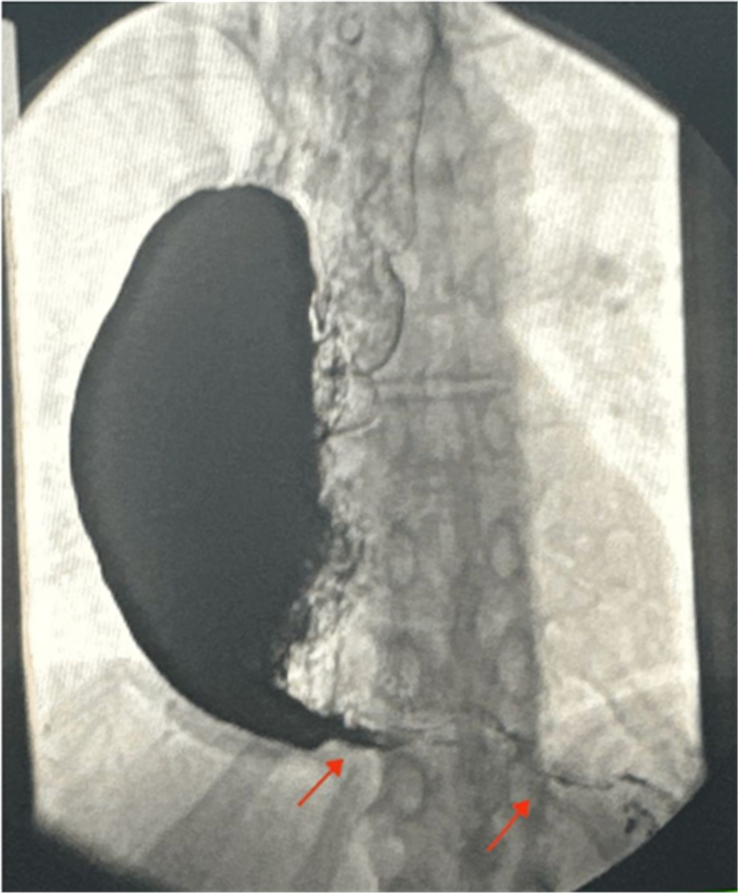
This image illustrates severe distal esophageal narrowing with delayed contrast emptying due to achalasia, while the gastroesophageal junction remains patent, allowing contrast flow into the stomach and small bowel (red arrows).

An upper gastrointestinal series was significant for an abrupt beak-like narrowing of the distal esophagus with retention of a large amount of barium in the distal esophagus and patulous dilation of the thoracic esophagus, as seen in Figure 1, similar to that seen in CT. The gastroesophageal junction is patent, with delayed antegrade progression of oral contrast into the stomach and proximal small bowel. Furthermore, an esophagogastroduodenoscopy was conducted and revealed entire dilation of the esophagus, an epiphrenic diverticulum, pooling of fluid, and evidence of food stasis without resistance to the passage from the esophagus to the stomach. Lastly, high-resolution manometry (HRM) showed unremarkable findings of normal lower esophageal sphincter pressure of 6 mmHg and 0% random peristaltic esophageal contractions.

The patient’s relentless symptoms and compelling imaging findings prompted the decision to proceed with an esophagectomy. The surgical team performed a robotic-assisted thoracoabdominal esophagectomy with gastric pull-through and jejunostomy tube placement. To prevent postoperative gastroesophageal reflux (GER), Dor fundoplication was performed without cutting the short gastric artery and vein. During the procedure, a conversion to an open right thoracotomy became necessary to facilitate the anastomosis between the remaining portion of the esophagus and the stomach. This intricate surgery proved to be technically challenging due to the patient’s history of multiple previous procedures, extensive adhesions, and a dilated esophagus.

Upon careful examination of the esophagectomy specimen, the pathologic findings revealed chronic inflammation with plasmacytosis, lymphoid aggregates, and fibrosis, which were identified as consistent with the underlying diagnosis of achalasia. These pathological findings further validate the necessity of surgical intervention. Postoperatively, the patient experienced many complications. New acute limb ischemia of right lower extremity with decreased sensation, motor function, and poikilothermia. Additionally, the patient suffered new-onset atrial fibrillation with rapid ventricular rate and thoracic duct injury with a milky bilateral pleural effusion. The management of these complications necessitated a collaborative, multidisciplinary approach. Additionally, an accordion drain was placed to collect the mediastinal fluid and antiarrhythmic therapy to manage atrial fibrillation. The patient was then transferred to the ICU for an emergent thrombectomy of the right lower extremity by a vascular surgery team. Postoperative recovery and long-term outcomes were closely monitored to assess the effectiveness of the esophagectomy in providing relief from the debilitating symptoms and improving his overall well-being. These complications, while challenging, underscore the importance of a comprehensive and coordinated approach to patient care, ensuring that the benefits of the surgical intervention are maximized and any adverse events are promptly addressed.

## Discussion

Achalasia is an uncommon disorder caused by the failure of the lower esophageal sphincter and a marked absence of peristalsis in the esophagus, leading to a functional obstruction of the GEJ^3^. A standard surgical method and an effective way to treat esophageal achalasia after gastric surgery is laparoscopic Heller myotomy with Dor fundoplication^8^. Patients can present with a whole array of symptoms, including chest pain, vomiting, and esophageal spasms. Occasional white and foamy vomit upon waking up is not uncommon in patients experiencing acid reflux frequently^9^. Esophageal spasms occur in patients with recurring gastroesophageal reflux because the stomach acids modify the afferent nerves of the peristaltic peripheral pathways, which leads to distal esophageal spasms^10^. The outcomes are postoperative GER and symptoms of dysphagia. Half of the cases of achalasia present with chest pain as a common symptom, and patients report improvement or complete resolution of chest pain after the procedure. Studies have shown that all patients who presented with chest pain before surgery reported either complete resolution or improvement of chest pain after undergoing surgery^11^. Pseudoreflux, a condition resembling reflux that presents as heartburn due to food stagnation and fermentation, which leads to a lowering of pH, has been reported in about 58.2% of individuals before the surgery, as stated in studies^11,12^. Approximately 21.8% of patients experience heartburn after the surgery^11^. Without objective pH, the exact prevalence of gastroesophageal reflux disease (GERD) remains elusive. Impaired acid reflux sensing, which is linked to neuropathic abnormalities, leads to a limited correlation between acid reflux and heartburn symptoms in achalasia^13^. The need for reoperation for redo fundoplication is obviated by proton pump inhibitors, which effectively manage the reflux symptoms^11^.

An undisputed preferred approach in current surgical practice is the inclusion of partial fundoplication, which is also regarded as the gold standard^9^. Robust clinical evidence, including a double-blind, randomized trial comparing myotomy alone to myotomy with Dor fundoplication, reveals a remarkable ninefold reduction in the risk of pathologic gastroesophageal reflux due to the addition of fundoplication^14^. The benefits of combining fundoplication with laparoscopic myotomy were strongly demonstrated in a comprehensive meta-analysis that comprised 7855 patients^11^. This study has unequivocally shown that the absence of fundoplication is associated with a 31.5% incidence of postoperative GERD, which was substantially reduced to 8.8% with its incorporation^11^. The unequivocally preferred choice is partial fundoplication, as opposed to Nissen fundoplication, due to its lower recurrence rates of dysphagia^11^.

Given its added benefit of reinforcing the exposed esophageal mucosa, which can be beneficial in cases of inadvertent mucosal perforation or mucosal thermal injury that was undiagnosed^7^, Siow *et al*.^11^ demonstrated in their study that surgeons in Malaysia more frequently favored Dor fundoplication. When it comes to selecting the type of partial fundoplication, research has demonstrated that there is no substantial difference between laparoscopic Dor and Toupet fundoplication in terms of relieving dysphagia and controlling postoperative acid exposure^15^. Several studies have indicated a notable degree of patient satisfaction scores with minimal complications^11^. This reflects a positive impact on the quality of life.

## Conclusions

This case of refractory achalasia highlights the persistent challenges of managing this rare esophageal disorder, especially in patients with extensive surgical histories. Despite prior surgeries, the patient’s symptoms persisted, emphasizing the relentless nature of achalasia. This case underscores the evolving landscape of achalasia management with emerging techniques such as POEM. Individualized surgical choices, considering the patient’s surgical history, remain crucial. This report contributes to our understanding of refractory achalasia, emphasizing the significance of a multidisciplinary approach, meticulous planning, and ongoing research for improved management.

## Ethical approval

Not applicable.

## Consent

Written informed consent was obtained from the patient for publication and any accompanying images. A copy of the written consent form is available for review by the editor-in-chief of this journal upon request.

## Source of funding

No funding was used in this study.

## Author contribution

F.K. and S.W.A.: writing – original draft; S.S.: data curation and formal analysis; R.S.: formal analysis and investigation; T.H.: investigation and resources; M.Y.S.: supervision and validation; U.A., T.Z., Z.S.R., A.M.K., and M.S.: data collection, data analysis or interpretation, and writing of the paper.

## Conflicts of interest disclosure

The authors declare no conflicts of interest.

## Research registration unique identifying number (UIN)

Not applicable.

## Guarantor

Mansi Singh, e-mail: singhmansi57@gmail.com.

## Data availability statement

The datasets used and/or analyzed during the current study are available from the corresponding author upon reasonable request.

## Provenance and peer review

Not commissioned, externally peer-reviewed.
